# Rainfall-driven dynamics of faecal pollution in river bathing sites: coupling hydrological events, and DNA-assisted tracking to improve pollution management in recreational waters

**DOI:** 10.3389/fmicb.2026.1796625

**Published:** 2026-08-10

**Authors:** Isabel Douterelo, Steve Juggins, Laura Díaz Garcia, Carlos Rubio Paniagua, Isabel Volz, Rick Battarbee

**Affiliations:** 1 School of Mechanical, Aerospace and Civil Engineering, University of Sheffield, Sheffield, United Kingdom; 2 School of Geography, Politics & Sociology, Newcastle University, Newcastle upon Tyne, United Kingdom; 3 School of Chemical, Materials and Biological Engineering, University of Sheffield, Sheffield, United Kingdom; 4 Environmental Change Research Centre, University College London, London, United Kingdom

**Keywords:** bacterial community, bathing water quality, citizen science monitoring, microbial source tracking, river management strategies, stormwater impacts

## Abstract

This study assesses the use of Microbial Source Tracking (MST) alongside 16S rRNA gene sequencing to overcome the limitations of Faecal Indicator Bacteria (FIB) in rivers by enabling source attribution of faecal pollution, improving microbial risk assessment, and supporting more effective bathing water management. During the 2023 bathing season, weekly samples were collected by citizen scientists at two River Wharfe sites: the Cromwheel, the Environment Agency’s (EA) bathing monitoring site upstream of the Ilkley Sewage Treatment Works (STW), and the Stepping Stones, downstream of the STW. Samples for DNA analysis were taken concurrently with the EA statutory FIB monitoring. The EA measured water physico-chemical parameters and modelled rainfall upstream in the catchment. DNA-based analyses included Microbial Source Tracking (MST) using droplet digital-PCR (ddPCR) to quantify general, human, and ruminant *Bacteroidales* markers, alongside 16S rRNA sequencing for bacterial characterisation. Rainfall was the main driver of FIB and *Bacteroidales* marker variation, with higher FIB correlated with 12 h cumulative antecedent rainfall at Cromwheel. At Cromwheel, human and ruminant sources were detected during dry weather, but wet weather *E. coli* peaks were dominated by human markers. At Stepping Stones, the human-associated marker was predominant, as expected for a site downstream of a STW, and correlated with elevated FIB and increased abundance of potential pathogens, including *Mycobacterium, Aeromonas* and *Clostridium*. The combined ddPCR-MST and sequencing approach enhanced the detection of low-level faecal pollution, distinguished sources, and revealed microbial community shifts associated with pollution. These results demonstrate the added value of molecular methods to inform recreational river management.

## 1 Introduction

Wild swimming surged in popularity during the COVID-lockdown, and more rivers in the UK are currently used now as recreational sites. Recreational waters, such as bathing areas, are particularly vulnerable to contamination, potentially exposing swimmers to harmful pathogens (Stec et al., 2022). High levels of faecal pollution have been directly related to disease outbreaks in recreational swimming areas, and it has been estimated that it causes 170 million enteric and respiratory illnesses annually worldwide (Hynes et al., 2024). Most rivers in England and Wales are affected by faecal pollution originating from different sources, ranging from sewer infrastructure to agricultural runoff, and this can cause a significant threat to river ecology and to public health if not managed properly (River Trust, 2025; Environment Agency SWIMINFO, 2025).

The River Wharfe is a popular recreational river located in The Yorkshire Dales (United Kingdom), where poor river water quality at a bathing site in the town of Ilkley was reported by the Ilkley Clean River Group (ICRG) (Battarbee et al., 2021). Following a successful campaign by the ICRG the site was designated by the UK Government in 2020 for bathing and became the first running water site in the country to be designated (DEFRA, 2020). However, statutory monitoring for faecal indicator bacteria (FIB) by the Environment Agency (EA) each year since 2021 indicates that the bathing site does not meet the minimum standards for safe bathing (Environment Agency, 2024b). The legal standards for *E. coli* and intestinal enterococci are defined using the 95th or 90th percentile of samples collected over a four-year period, with thresholds for good quality status, in inland waters set at 1,000 and 400 cfu/100 mL (95th percentile), respectively (The Bathing Water Regulations 2013). Now, the local community in Ilkley is collaborating with researchers, the local water company (Yorkshire Water) and the EA in a major project to improve water quality in the river along the bathing water, both upstream and downstream of the town’s wastewater treatment plant (WWTP). From previous studies of the bathing water site, and sites in the catchment upstream (Karunakaran et al., 2024) it was evident that reducing faecal pollution to meet the required standard for bathing would be challenging due to the amalgamation of different contamination sources in the river. During wet weather, spills from Combined Sewer Overflows (CSOs) can discharge rainwater mixed with untreated sewage that contains pathogens and can cause illness (Schellart et al., 2025). However, faecal sources of pollution also include discharges from WWTPs, pumping station overflows (PSOs), septic tanks, misconnections in pipes, runoff from agricultural land with manured fields as well as faeces from livestock and wildlife. It is crucial to assess the relative importance of these different sources accurately in order to protect water quality and ensure public safety.

Traditional faecal pollution monitoring approaches that often rely on infrequent sampling of FIB, do not adequately capture the dynamic nature of pollution events and their wider impact on river status. The FIB approach, used to estimate faecal pollution in water, relies on culturing and counting coliform bacteria (*E. coli*) and intestinal enterococci (IE). While it is useful for stakeholders to assess regulatory compliance, this 150-year-old culture-based method has limitations. It only quantifies a few indicator species, does not reflect the full diversity of pathogens, and often only weakly correlates with actual pathogen presence (Holcomb and Stewart, 2020). Critically moreover, FIB counts do not identify and quantify pollution sources (e.g., human, agricultural and wildlife). As an alternative, DNA-based source tracking techniques, by analysing unique genetic markers, can identify contamination sources and their relative contributions to pollution loads. Furthermore, an emerging technology, digital droplet PCR (ddPCR) enables the quantification of nucleic acids independently of a standard reference material and provides an absolute rather than relative quantification of the targeted genes. ddPCR assays are overall more robust when compared with traditional qPCR approaches, are less compromised by environmental inhibition and face fewer issues relating to substrate competition, enabling a more effective use of multiplex assays (Staley et al., 2018). Similarly, Microbial Source Tracking (MST) tools based on microbial community profiling using DNA-based amplicon sequencing have been developed to allocate sources of pollution. Microbial community profiling to establish distinctive bacterial community signatures, has been successfully applied to trace and differentiate faecal pollution in aquatic environments (Henry et al., 2016; Unno et al., 2018).

The primary objective of this study was to combine environmental monitoring with advanced molecular analysis using data from the River Wharfe, to demonstrate how a molecular-based monitoring program could be designed to identify sources of pollution at designated bathing water sites. This approach can also uncover genetic information in a way that the threats to public health could be better understood and managed. The information obtained is of special value to stakeholders, including water utilities needing to invest in infrastructure projects required to improve river water quality for bathing.

## 2 Materials and methods

### 2.1 Study sites and sample collection

**Figure 1:**
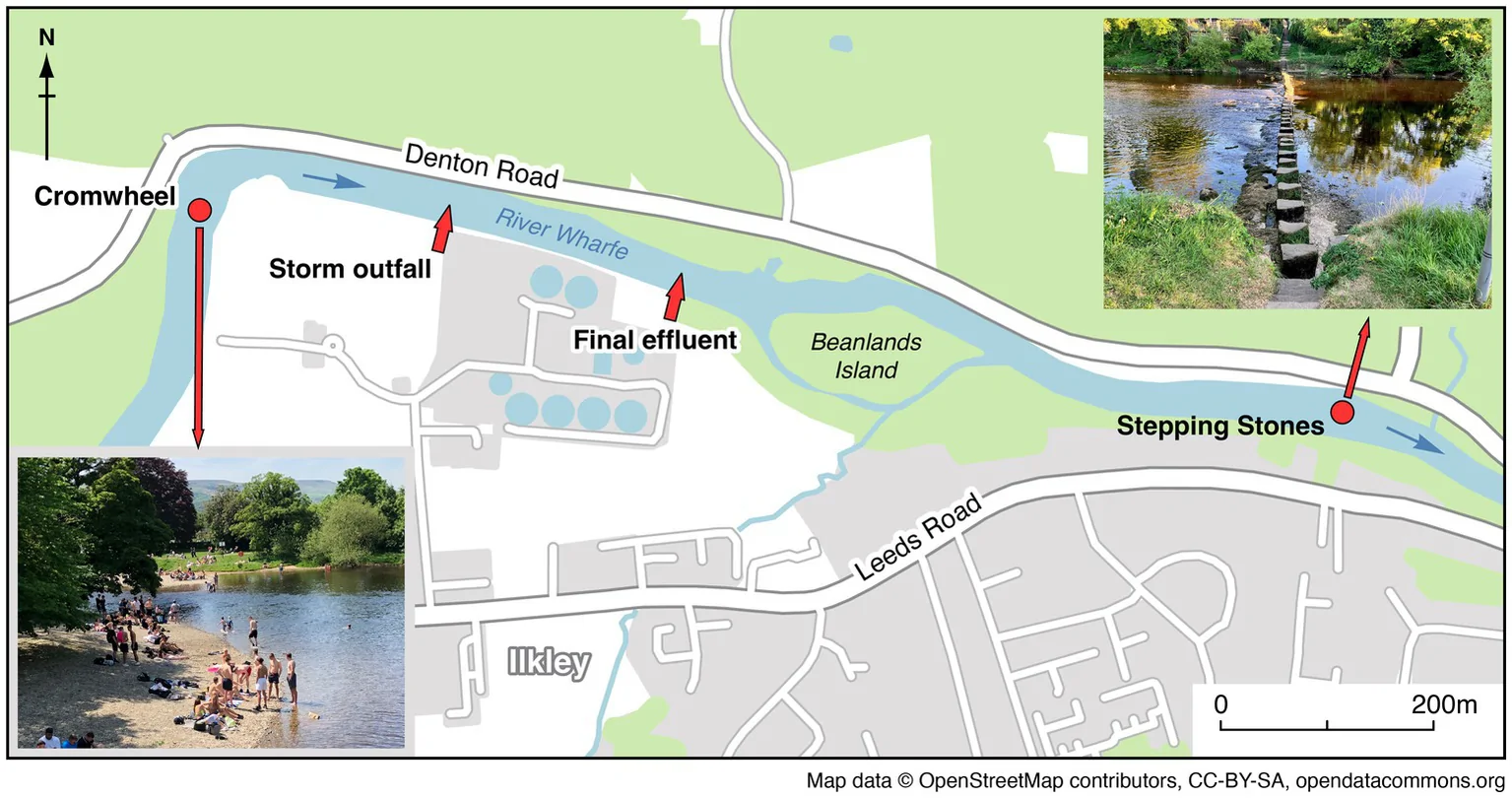
Sampling sites at the River Wharfe, showing the designated bathing site known as the Cromwheel upstream of Ilkley WWTP, and the Stepping Stones site downstream of the WWTP.

To assess bathing water quality at Ilkley (West Yorkshire, United Kingdom), fieldwork was carried out on the River Wharfe, sampling both upstream and downstream of the newly designated bathing water site known as the Cromwheel (Figure 1). Land use in the catchment is dominated by grassland and pastoral agriculture, mainly sheep and cattle farming. Improved grasslands are concentrated in the valley bottom, giving way to semi-natural grassland and peat moorland at higher elevations. Woodland cover is relatively limited, accounting for 5.1% of the catchment (Environment Agency, 2024a). Two settlements in the area, Addingham and Ilkley are served by Ilkley WWTP which discharges downstream of the Cromwheel bathing water.

In a previous study, the river was sampled at various points, including the main river and tributaries, and faecal contamination was detected along the river (Karunakaran et al., 2024). Based on this previous study, we have focused on two specific sites: Cromwheel and Stepping Stones. The samples were taken at the same time and place as samples collected by the EA for statutory faecal bacteria monitoring under the UK’s Bathing Water Regulations. The upstream site, the “Cromwheel” site, is the bathing water monitoring point used by the EA. The downstream site, Stepping Stones, is a secondary site used to assess the impact of effluent from the WWTP. By co-locating our samples with those of the EA we were able to use EA data in statistical analyses and make direct comparisons between the EA’s data for FIB (*E. coli* and IE) and our DNA-based data for the presence and identity of faecal pathogens. The two sites were sampled 19 times by citizen scientists, members of the Ilkley Clean River Group and the Addingham Environment Group. The two sites were sampled weekly, from May to September 2023 and all samples were collected from 11:30 to 14:30. The collected samples were transported within 24 h to The University of Sheffield for filtering and subsequent DNA extraction.

### 2.2 Physico-chemical monitoring and Faecal Indicator Bacteria (FIB)

Water quality parameters including alkalinity, pH, temperature, conductivity, dissolved oxygen and nutrients (e.g., ammonia, nitrate, nitrite and orthophosphate) were measured by the EA using spot samples collected weekly at the time when samples were collected for DNA-based molecular analysis. All the physico-chemical analyses were carried out according to the Standing Committee of Analysts, (SCA) methods (Standing Committee of Analysts., 1976, *Methods for the examination of waters and associated materials*, https://www.standingcommitteeofanalysts.co.uk/). The EA also provided modelled antecedent catchment rainfall data, river level and FIB data [*E. coli* and Intestinal Enterococci (IE)] analysed using current standard methods (ISO9308-1 and ISO7899-2, respectively).

### 2.3 Water samples collection and filtration for DNA analysis

All water samples for DNA analysis were collected in sterile polyethylene bottles, immediately placed on ice in dark cooling boxes, and processed by filtering within 24 h of collection. 500 mL of sample was filtered through 0.2 μm sterile nitrocellulose membrane filters (Sartorius, United Kingdom). Three replicates were obtained per sample point and date, giving a total of 120 filters, which were preserved in sterile bags at −80°C for subsequent DNA extraction. DNA was extracted from the filters using a Qiagen Power soil extraction kit (Qiagen, United Kingdom). The quantity and purity of the extracted DNA (260/280 ratio) were measured using Nanodrop ND-1000 spectrophotometer (NanoDrop, Wilmington, DE). For subsequent amplicon sequencing and ddPCR analysis, triplicate samples were pooled.

### 2.4 Droplet digital PCR (ddPCR) for quantitative microbial source tracking (qMST): host-specific *Bacteroidales* markers

One of the most widely used MST approaches utilises PCR to quantify host-specific microorganisms, using *Bacteroidales* markers to discriminate between human and other animal faecal sources (Malla et al., 2024). A total of 40 DNA samples were analysed by ddPCR to obtain representative samples of both sites potentially affected by faecal pollution. All the samples were run in duplicate, including a positive control (DNA from a STW in Yorkshire), and faecal material from ruminants [pooled from faeces from sheep, cow, pig, goat and horse obtained from Graves Park Animal Farm (Sheffield, United Kingdom)]. No-template controls, PCR master mix containing nuclease free water instead of sample DNA, were also included to verify the lack of contamination. The *Bacteroidales* oligonucleotide sequences used in the ddPCR assays are described in Supplementary Table S1.

A duplexed PCR master mix targeting HF183 and the GenBac3 gene was created by adding 0.9 nM of the respective forward and reverse primers, 0.25 nM of the respective probes, 12.5 μL of ddPCR™ 2X Supermix for Probes (nodUTP, Bio-Rad Laboratories United Kingdom), 5 μL of DNA (2 ng/μl), and nuclease free water to a final reaction volume of 22 μL. The ruminant assay BacB2 was run independently using the same reagents, concentrations and volumes but with different primers and probe combinations. Each sample was run in duplicate. Primers and probes were purchased from Eurofins Scientific (Eurofins, United Kingdom). Positive and no template controls were included with each assay.

The PCR plate was placed in a C1000 Touch™ Thermal Cycler (Bio-Rad) and amplification was performed with the following temperature profile: 10 min at 95°C for initial denaturation, 40 cycles of 95°C for 30 s, and 58°C for 60 s with a ramp rate of 2°C/s, followed by 98°C for 10 min, then an indefinite hold at 4°C. The amplicons were then placed in a QX200™ instrument (Bio-Rad) and droplets were analyzed according to manufacturer’s instructions for 6-FAM™/HEX™. Data acquisition and analysis were performed with QuantaSoft™ v. 1.7 (Bio-Rad). The fluorescence amplitude threshold, distinguishing positive from negative droplets, was set manually for each marker gene. The same threshold was applied to all the wells. QuantaSoft uses the Poisson distribution to quantify the concentration of targets based on the numbers of positive and accepted droplets in each well. Samples were quantified in duplicate and replicate wells were merged. A sample was considered positive and quantifiable if the minimum threshold of positive droplets was met.

### 2.5 16S rRNA gene sequencing to obtain community fingerprints

To obtain representative samples to analyse bacterial diversity through sequencing of the 16S rRNA gene, 40 samples representing each site and date were used to obtain a bacterial fingerprint over time. Samples were sequenced at Mr. DNA (Shallowater, TX, United States), using a MiSeq platform following the manufacturer’s guidelines. A fragment of the 16S rRNA gene was sequenced by using primers 28F and 519R spanning the V1 to V3 region. PCR conditions and subsequent sequencing methods follow Karunakaran et al. (2024). Bioinformatics analysis based on ZOTUs (Zero-radius Operational Taxonomic Units) or ASVs (Amplicon Sequence Variants) was performed to distinguish sequences with even a single nucleotide difference. Briefly, raw sequences were depleted of primers, short sequences < 150 bp and sequences with ambiguous base calls were removed. Sequences were quality filtered using a maximum expected error threshold of 1.0 and dereplicated. The dereplicated or unique sequences were denoised, followed by chimera removal, thereby providing a denoised sequence or zOTUs. Final zOTUs were taxonomically classified using BLASTn against a curated database derived from NCBI^1^.

### 2.6 Statistical analysis

The significance of differences in chemistry between sites was assessed using generalised least squares (GLS) with a first-order autoregressive correlation structure to account for temporal autocorrelation (Tasker and Stedinger, 1989). Differences between sites were considered significant when the *p*-value was <0.05.

Patterns of variation in 16S rRNA bacterial community composition at the class level were explored using non-metric multidimensional scaling (nMDS; Minchin, 1987) with Bray-Curtis dissimilarities (Faith et al., 1987). Chemical and physical environmental variables were fitted as linear trend surfaces to nMDS axes to examine which variables were related to the main patterns of turnover in the bacterial data. Samples were also classified into groups with similar DNA composition using hierarchical cluster analysis of Bray-Curtis dissimilarities using Ward’s method (Murtagh and Legendre, 2014). The number of groups was determined using a combination of average silhouette width and species fidelity measures (Borcard et al., 2018).

Non-parametric Spearman correlation between physico-chemical, environmental and microbiological parameters using data from both Cromwheel and Stepping Stones was run for each site independently. All analysis was performed using R version 4.5.1 (R Core Team, 2025).

## 3 Results

### 3.1 Water quality monitoring

**Figure 2:**
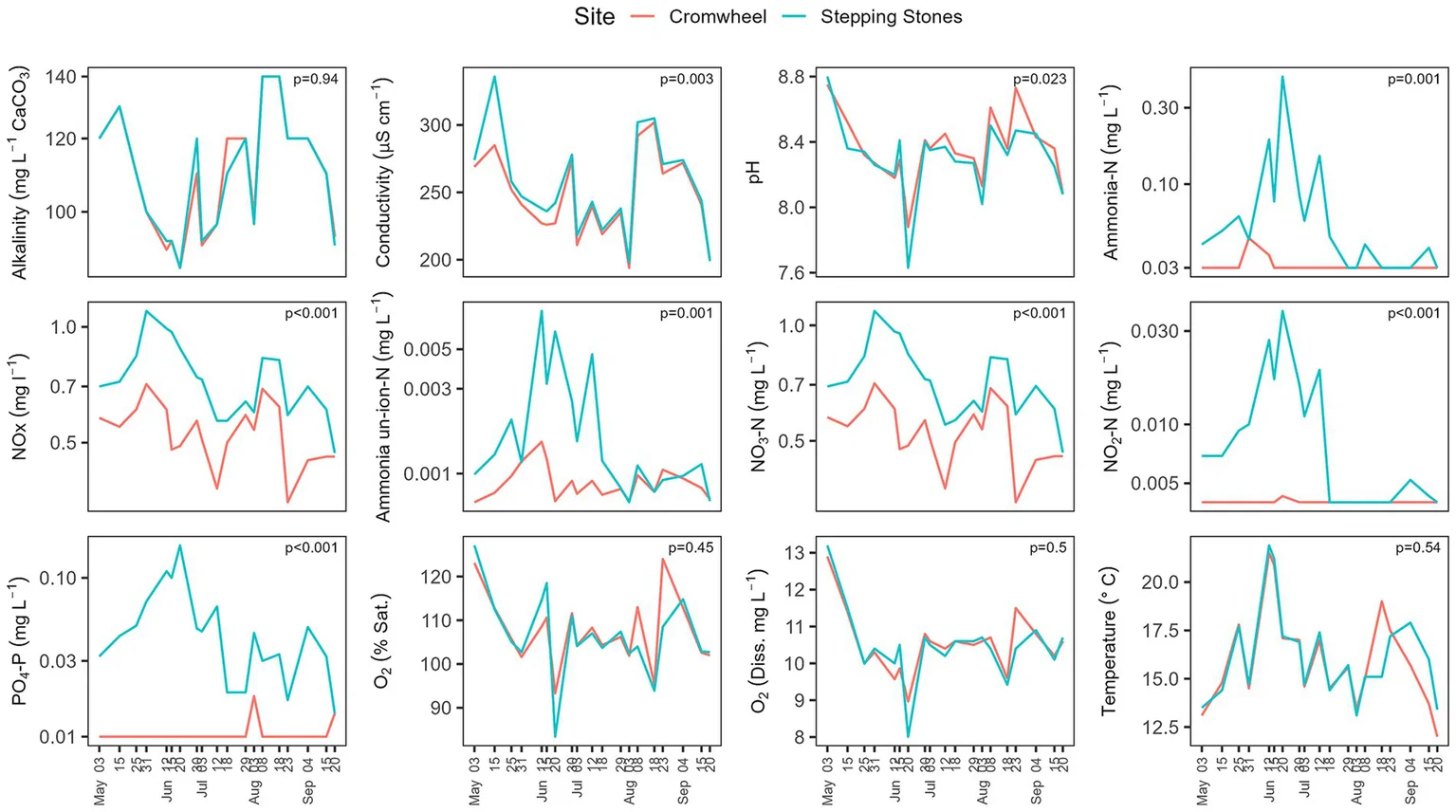
Comparison of physico-chemical parameters measured in water samples from the Cromwheel and Stepping Stones sampling sites, illustrating spatial differences between the two locations. *p*-values are indicated at the left top corner of each individual graph; the differences were considered significant when the *p*-value was <0.05. NOx represents the sum of nitrate (NO_3_) and nitrite (NO_2_).

Results from the physico-chemical analysis of water samples are shown in Figure 2. The water temperature for both sites ranged from low temperature in May of 13 °C-14 °C to highest temperature above 21 °C in June. pH remained relatively stable between 7.6 to 8.7 for the duration of the monitoring campaign at both sites. Conductivity values were similar at both sites, and ranged between 190 and 300 μS/cm. The percentage of dissolved oxygen saturation (DOsat) and dissolved oxygen (DO) was similar at both sites, ranging from 100 to 128%. The lower oxygen levels at both sites, below 100% DOsat, were on the 20th of June (93.3% for Cromwheel and 83.4% in Stepping Stones) and 18th of August (95.3% for the Cromwheel and, 93.9% at the Stepping Stones).

Regarding nutrients, nitrogen and phosphorus compounds, levels were clearly significantly higher (*p* ≤ 0.001) during the monitoring period at Stepping Stones. Ammonia (N) at the Cromwheel was 0.03 mg/L for most samples, except for the 31st of May (0.046 mg/L) and 12th of June (0.036 mg/L). The values of ammonia (N) for the Stepping Stones were higher than from the Cromwheel, with values between 0.042 and 0.063 mg/L for all the samples in May. The values of ammonia (N) reached a maximum of 0.34 mg/L the 12th of June and were lower in most of July and August samples, only the 8th of August (0.042 mg/L) values were higher. Similar trends were observed for un-ionised ammonia, reaching values in the Stepping Stones, the 12th of June (0.00821 mg/L), 20th of June (0.00629 mg/L) and 12th of July (0.00469 mg/L). Nitrate (NO_3_-N) was higher at the Stepping Stones, with values higher than 0.5 mg/L for all the samples analysed. Nitrite (NO_2_-N), as expected follows similar fluctuations to those observed for the other N compounds. Orthophosphate (PO_4_-P) was <0.01 mg/L for all days analysed at the Cromwheel, except on the 3rd of August when it reached 0.018 mg/L. The levels of orthophosphate were higher at the Stepping Stones, with values higher than 0.045 mg/L on 12th and 30th of June, and 3rd and 12th of July.

### 3.2 Antecedent rainfall and Faecal Indicator Bacteria (FIB)

**Figure 3:**
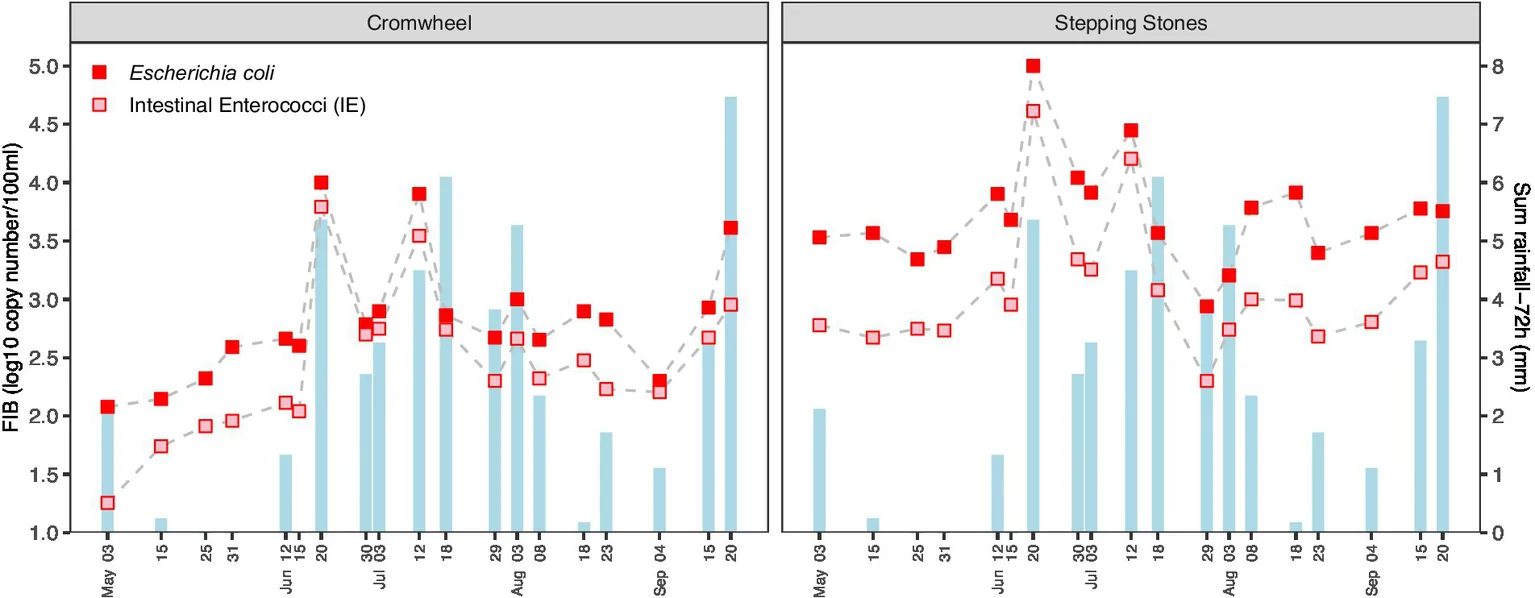
Comparison of faecal indicator bacteria (FIB) concentrations, *Escherichia coli* and intestinal enterococci (IE), with 72 h antecedent rainfall at the Cromwheel and Stepping Stones sampling sites, illustrating relationships between rainfall and microbial water quality.

The association between antecedent rainfall and FIB levels was analysed during the monitored period. Figure 3 shows the results for both sampling sites, expressed as log10 cfu/100 mL. Overall, the levels of FIB tended to be higher at the Stepping Stones than at the Cromwheel, with *E. coli* values > 2.5 log10 cfu/100 mL for all the samples and IE > 1.5. log10 cfu/100 mL. At the Cromwheel, *E. coli* and IE values, log10 cfu/100 mL, were below 3 for most of the sampling times. Maximum values (>4 for *E. coli* and > 3.5 for IE) were reached on the 20th of June, coincident with a heavy antecedent rainfall event (sum-rainfall-72 h = 5.36 mm). On the 12th of July, *E. coli* values also reached > 4 and > 3.5 coincident again with a heavy rainfall event sum-rainfall-72 h (4.50 mm). Another peak was observed on the 20th of September with *E. coli* >3.5 and IE > 2.5. These values were coincident with cumulative rainfall-72 h of 7.47 mm.

At the Stepping Stones, FIB values were consistently higher than at the Cromwheel, with *E. coli* values between 3 and 5 log10 cfu/100 mL and IE between 2 and 4.5 log10 cfu/100 mL, for most sampling dates. Maximum values were reached on the 20th of June: *E. coli* 5 and IE 4.5 log10 cfu/100 mL coincident with accumulative-rainfall-72 h (5.36 mm). On the 12th of July higher concentrations were also observed *E. coli* 4.5- and IE 4 log10 cfu/100 mL with values of sum-rainfall 72 h (4.50 mm).

Both sites fail to meet the minimum standards for safe bathing. Whereas this is expected at the Stepping Stones, given its location immediately downstream of the WWTP, the reason for the failure at the Cromwheel site is less certain as potential sources of pathogenic bacteria upstream of the WWTP due to surface water run off include livestock (cows and sheep), wildlife, birds and dogs, as well as humans.

### 3.3 ddPCR with general, ruminant and human *Bacteroidales* specific markers

**Figure 4:**
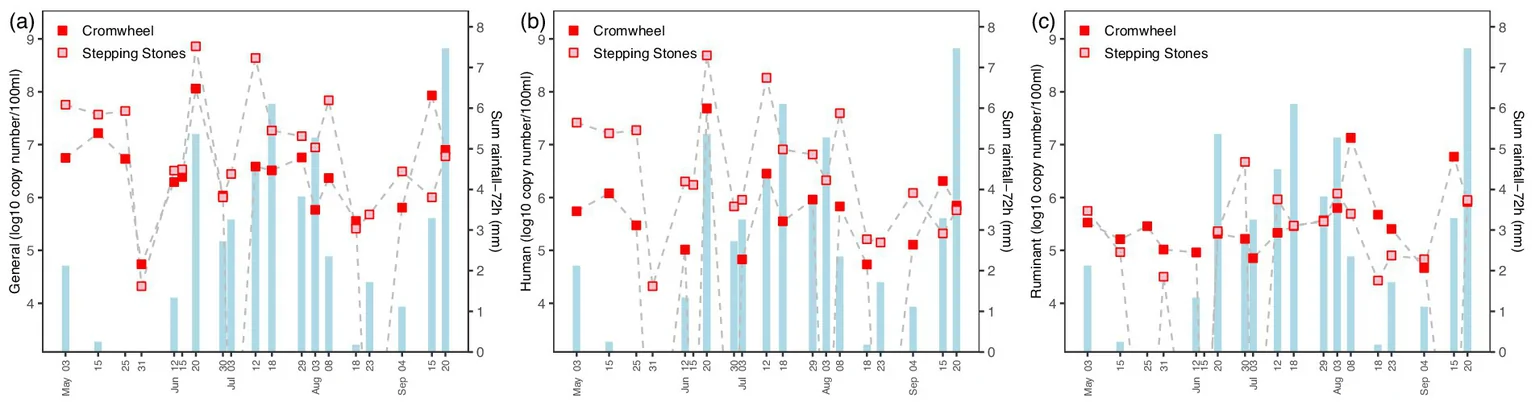
ddPCR results for *Bacteroidales* markers: **(a)** general (GenBac3), **(b)** human associated (HF183) and **(c)** ruminant associated marker (BacB2) expressed in Log10 gene copy numbers/100 ml of sample. Blue vertical bars represent 72 h antecedent rainfall.

Figure 4 shows the results calculated as log10 of the gene copy numbers in 100 mL, for ddPCR regarding quantification of samples for the 3 genetic *Bacteroidales* markers, General *Bacteroidales* (GenBac3), Human-specific (HF183), and Ruminant-specific (BacB2). Overall, the presence of all these faecal markers was higher in the Stepping Stones than in the Cromwheel with the general marker (GenBac3) values above 3 for most of the samples except for the 20th of June and the 12th of July when values increased to above 5. Both days are associated with heavier rainfall events (Figure 4). The marker that clearly fluctuated, together with the general *Bacteroidales* (GenBac3) was the human-specific marker (HF183). This was also higher on rainy days (e.g., 20th of June) at both sampling sites and followed a similar trend over time. The ruminant marker (BacB2) showed a lower copy number for all samples at both sites (<4), but tended to increase with the other two markers, influenced by wet weather events. The level of the ruminant specific marker (BacB2) tended to be higher at the Cromwheel than at the Stepping Stones, although after rainfall both tended to increase.

### 3.4 Bacterial diversity 16S rRNA sequencing analysis

**Figure 5:**
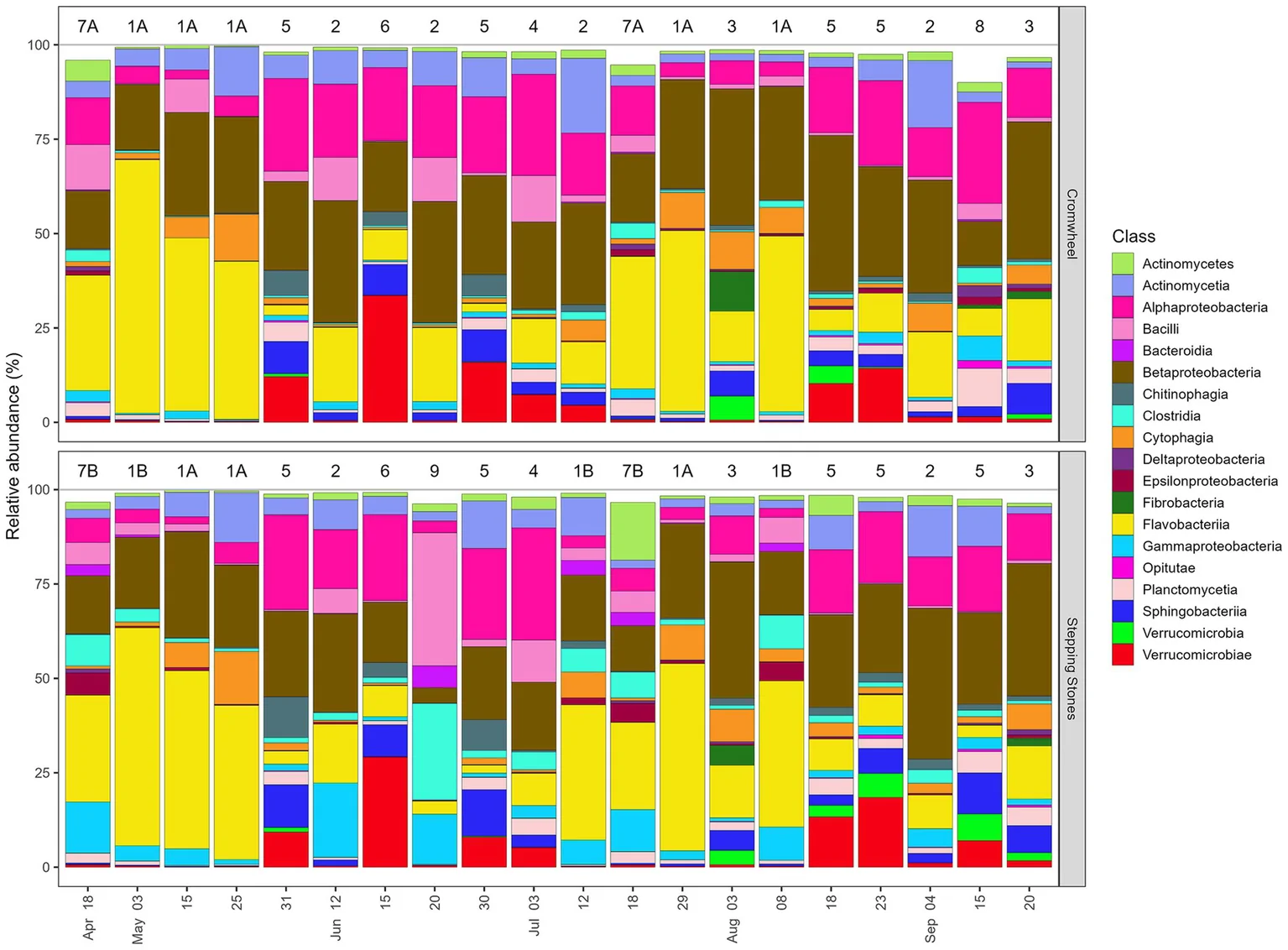
Percentage of relative abundance of bacteria at class level over time at both sites. The numbers and letters combinations on the top of each column indicates the nMDS cluster.

The amplicon sequencing analysis showed clear temporal variability of bacterial classes at both sites (Figure 5). The main class presented in all the samples were: Flavobacteria, Betaproteobacteria, Alphaproteobacteria and Actinomycetia. Flavobacteria were abundant particularly in May and July at both sites with percentages between 20 and 60%. Betaproteobacteria and Alphaproteobacteria, both increased in June (11–47%). Alphaproteobacteria had higher values >20% in the Cromwheel on samples from the 3rd of July, and 11th of September and Stepping Stones 31st of May, 15th of June and 3rd of July. Betaproteobacteria were present in most of the samples, but it was higher (>20%) in August and September for both sites. Other Proteobacteria, Gamma (<20%) and Epsilon (<10%), were present but with less relative abundance. Actinomycetia (<20%) was also present in all the samples, its values were higher in the Cromwheel samples from 12th July and 4th of September.

Other bacterial classes represented in most of the samples were Sphingobacteria, Planctomycetia and Cytophaga. The percentage of these groups together is not higher than 20% for all the samples. Sphingobacteria represented > 10% of the community in the Stepping Stones samples (31st of May, 30th of June 15-20th of September). With higher percentages, and on some dates only present in Stepping Stones, Bacteroidia represented 6% of the bacterial community abundance in 20th of June samples in the Stepping Stones. Clostridia increased together with Bacteroidia, showing how heavy rainfall clearly increased faecal pollution. Clostridia were also high on the 8th of August. The percentage of this type of bacteria was higher in the Stepping Stones when compared with the Cromwheel. Similarly, Bacilli that has small percentages increased on the 20th of June, coincident with a wet weather event.

Regarding classes commonly associated with animals’ intestinal tracts, Bacteroidia, Fibrobacteria, Planctomycetes and Clostridia were found in several samples but were higher in samples from the Stepping Stones in June and July, representing more than 25% of the total bacterial community on the 20th of June.

**Figure 6:**
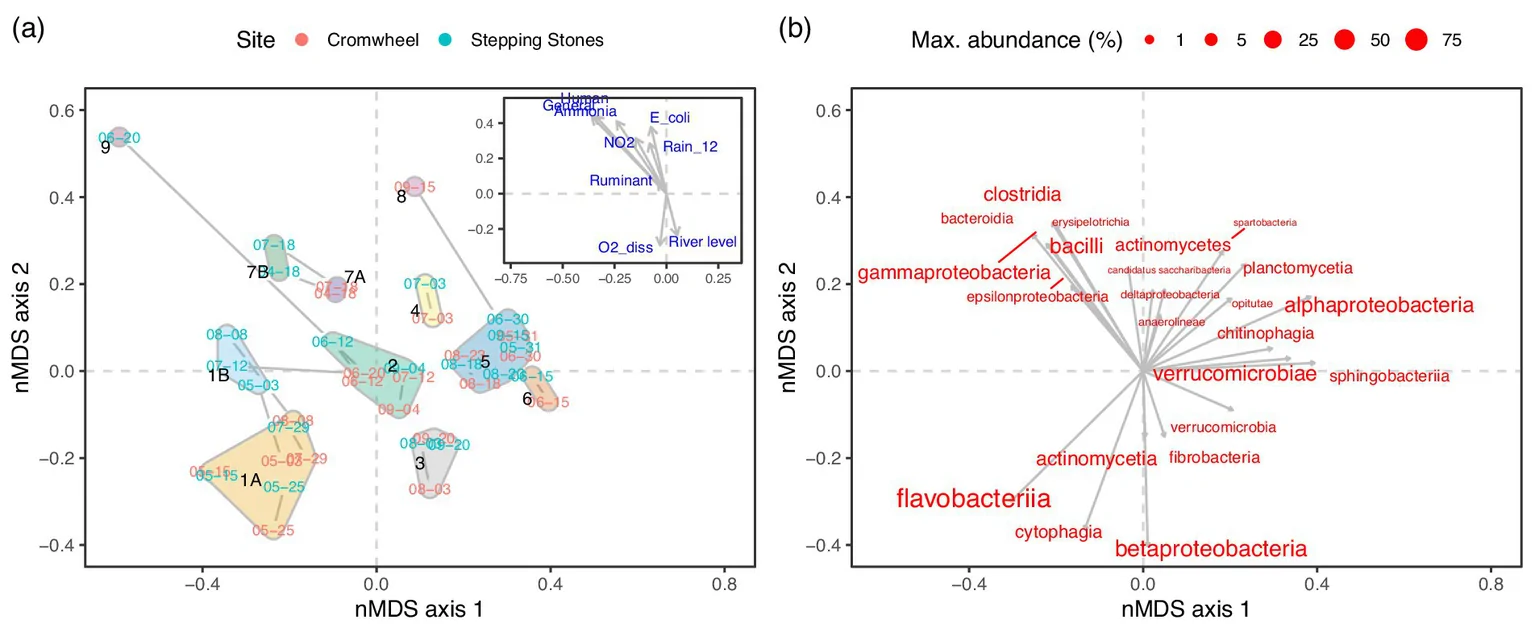
nMDS analysis, showing **(a)** position of samples, coded by date (month-day), with paired Cromwheel/Stepping Stones sample joined by gray line. Numbers and hulls indicate sample groups defined by hierarchical clustering. Inset shows direction of change in fitted physical, chemical and bacterial variables, and **(b)** directions of variation of 16S rRNA bacterial taxa.

Figure 6 shows the results of the nMDS ordination with sample groups identified by cluster analysis overlain. The stress value for the nMDS was 0.14, indicating a usable ordination (McCune and Grace, 2002), with clusters separated in ordination space. The biggest clusters were 1 and 5. Cluster 1 presented a high percentage of Flavobacteria, Actinomycetia, and Cytophagia related with high alkalinity and higher than average DO and pH. This group can be subdivided in 1A generally by low rainfall and lower flows, and 1B associated with higher rainfall events. Ammonium and *E. coli* were higher in 1B, with higher Cytophagia, Clostridia, Epsilonproteobacteria and Gammaproteobacteria. These were all from Stepping Stones samples downstream of the STW. Cluster 5 is characterized by the presence of Actinomycetia, Alphaproteobacteria, Sphingobacteria, Verrucomicrobiae, Chitinophagia and Planctomycetia. This cluster is defined by high alkalinity, low ammonium and *E. coli* levels higher at the Stepping Stones than for the Cromwheel samples. Generally, this cluster was also associated with low rainfall and low flows. Regarding the *Bacteroidales* faecal indicators, this cluster presented similar levels to Cluster 1 but has very low human (HF183) and General (GenBac3) bacterial markers, but similar *E. coli* levels (Figure 6).

**Figure 7:**
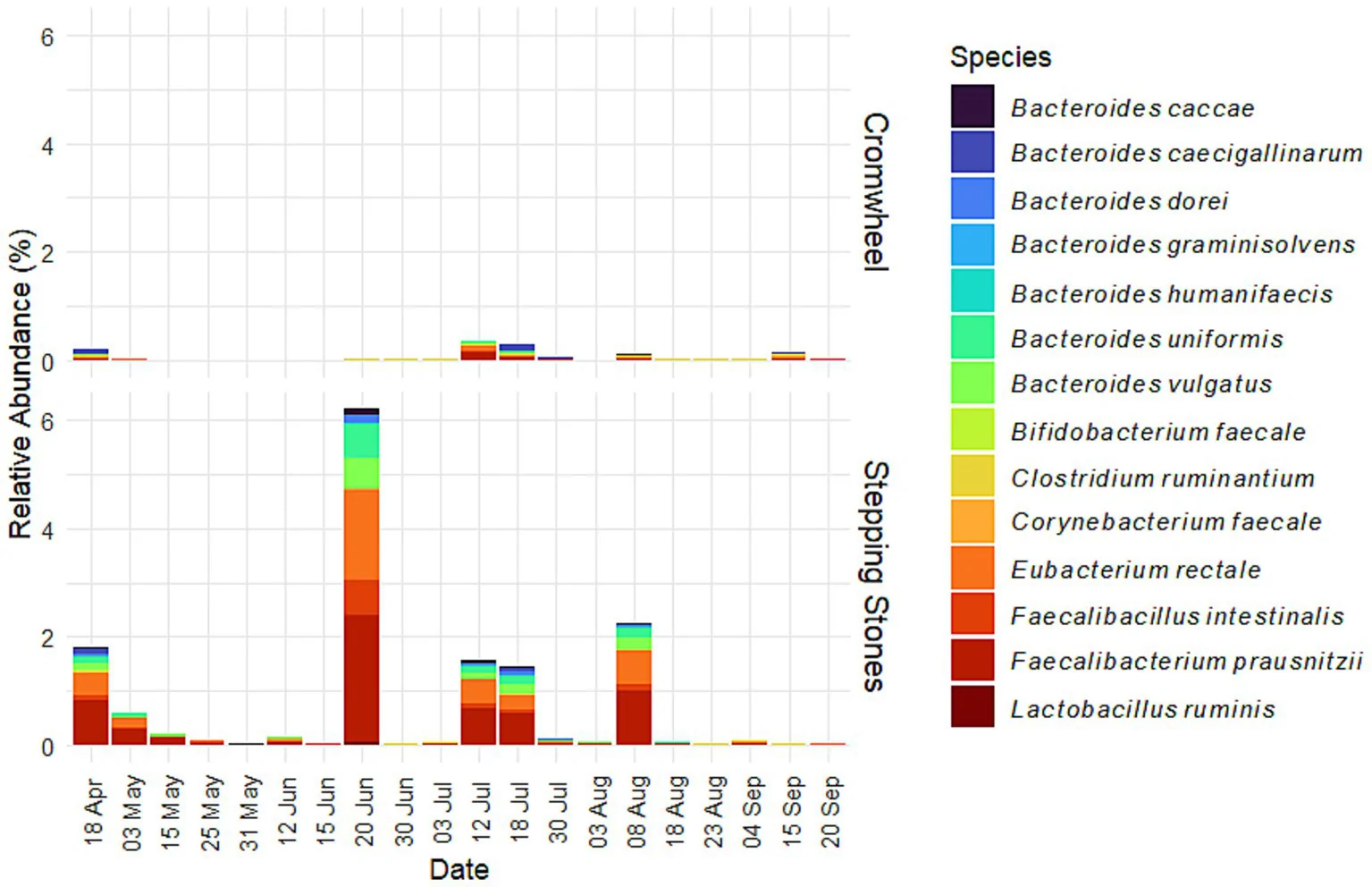
Assignment at species level of faecal indicator bacteria by 16S rRNA gene amplicon sequencing.

Cluster 3 is characterised by the presence of high Fibrobacteria, Verrucomicrobiae, Sphingobacteria, and Cytophagia. In relation to physico-chemical factors, this group was associated with low ammonium, alkalinity and temperature, but the highest river level and high rainfall average, together with high general faecal markers (GenBac3) and low human markers (HF183) for the Cromwheel samples. Clusters 2 and 3 were intermediate between 1 and 5. Cluster 6, the bacterial community composition, is similar to Cluster 5 except for very high abundance of Verrucomicrobiae. The physico-chemical factors determining the cluster were also resembling those in group 5, but associated with low alkalinity, low rainfall and river level and high temperature, indicating a dry period during sampling. Ruminant specific markers (BacB2) were very low, but average levels of General *Bacteroidales* (GenBac3) were associated with this cluster.

Cluster 7 was subdivided in 7A and 7B, with samples from the Cromwheel in 7A, and samples from Stepping Stones in 7B. Its composition is similar to Cluster 1, but with higher Flavobacteria, and Deltaproteobacteria, and very high Actinomycetia and Bacteroidia at the Stepping Stones. This cluster is associated with high rainfall and average levels of ammonium and *E. coli.* Cluster 8 is formed by a single sample from the Cromwheel linked to Cluster 5. This presented very high relative abundance of Planctomycetia, Alphaproteobacteria- and unusually high Anaerolineae, Candidatus, Deltaproteobacteria, Spartobacteria and Opitutae. Physico-chemical parameters were average, but higher than average ruminant specific (BacB2) levels. Cluster 9, comprises a single sample from Stepping Stones linked to Cluster 2, characterized by very high relative abundance of Bacilli and Clostridia, and unusually high levels of Erysipelotrichia and Bacteroidia. This sample is associated with very low alkalinity, very high ammonia, *E. coli* and high levels of general (GenBac3) and human associated (HF183) markers.

At genera level (Supplementary Figure S2) the most abundant genera are shown in a heatmap for all samples at both sites, including *Limnohabitans*, *Flavobacterium*, *Polynucleobacter* and *Rhodoferax.* All these bacteria tended to be more stable in Stepping Stones and varied showing more marked shifts in the Cromwheel sampling site over time. Other genera such as *Pseudomonas*, *Acinetobacter*, *Aeromonas* and *Hydrogenophaga* were also present at both sites, but showed less frequent and marked fluctuations in Stepping Stones, and more peaks at the Cromwheel in several samples, particularly in July and September. Faecal indicators including *Faecalibacterium* and *Bacteroides*, and potential pathogenic bacteria such as *Clostridium*, tended to also be more stable in Stepping Stones samples over time, showing higher fluctuation in samples at the Cromwheel coincident with increasing rainfall (e.g., 12th July and 15th September).

Several faecal indictor bacteria were found in the samples (Figure 6). Clearly, these were more abundant in Stepping Stones than in Cromwheel and increased associated with rainfall events. Overall, the relative abundance of these indicator bacteria was low, representing <6% of the total relative abundance in the samples. *Bacteroides* spp. are indicators of faecal pollution, and several species are human specific including: *Bacteroides dorei*, *Bacteroides uniformis* and *Bacteroides vulgatus*. Other faecal indicators such as *Faecalibacterium prausnitzii* and *Faecalibacterium intestinalis* (Martín et al., 2023) were also present in Stepping Stones (1–3%).

The abundance of *Bacteroides graminisolvens* (<0.2%) and *Clostridium ruminantium*, host-specific bacteria of ruminants (Reid et al., 2017), was very low (relative abundance <1%) at both sites. *Bacteroides caecigallinarum*, has been isolated in chicken caecum and can show the contribution of birds to the overall faecal pollution (Kollarcikova et al., 2020). This shows the potential of amplicon sequencing in being used to indicate faecal pollution sources.

At species level, several pathogens and opportunistic pathogenic taxa were found in the samples (Supplementary Table S2), such as *Aeromonas* spp., *Shigella* spp., and *Enterococcus* spp. All of these were found at very low relative abundance in all the samples, and overall, more abundant in the Stepping Stones samples than in the Cromwheel ones, except for *Aeromonas hydrophila*, *Helicobacter heilmannii*, and *Leptospira* spp.

### 3.5 Correlation between physico-chemical and microbiological parameters at both sites

**Figure 8:**
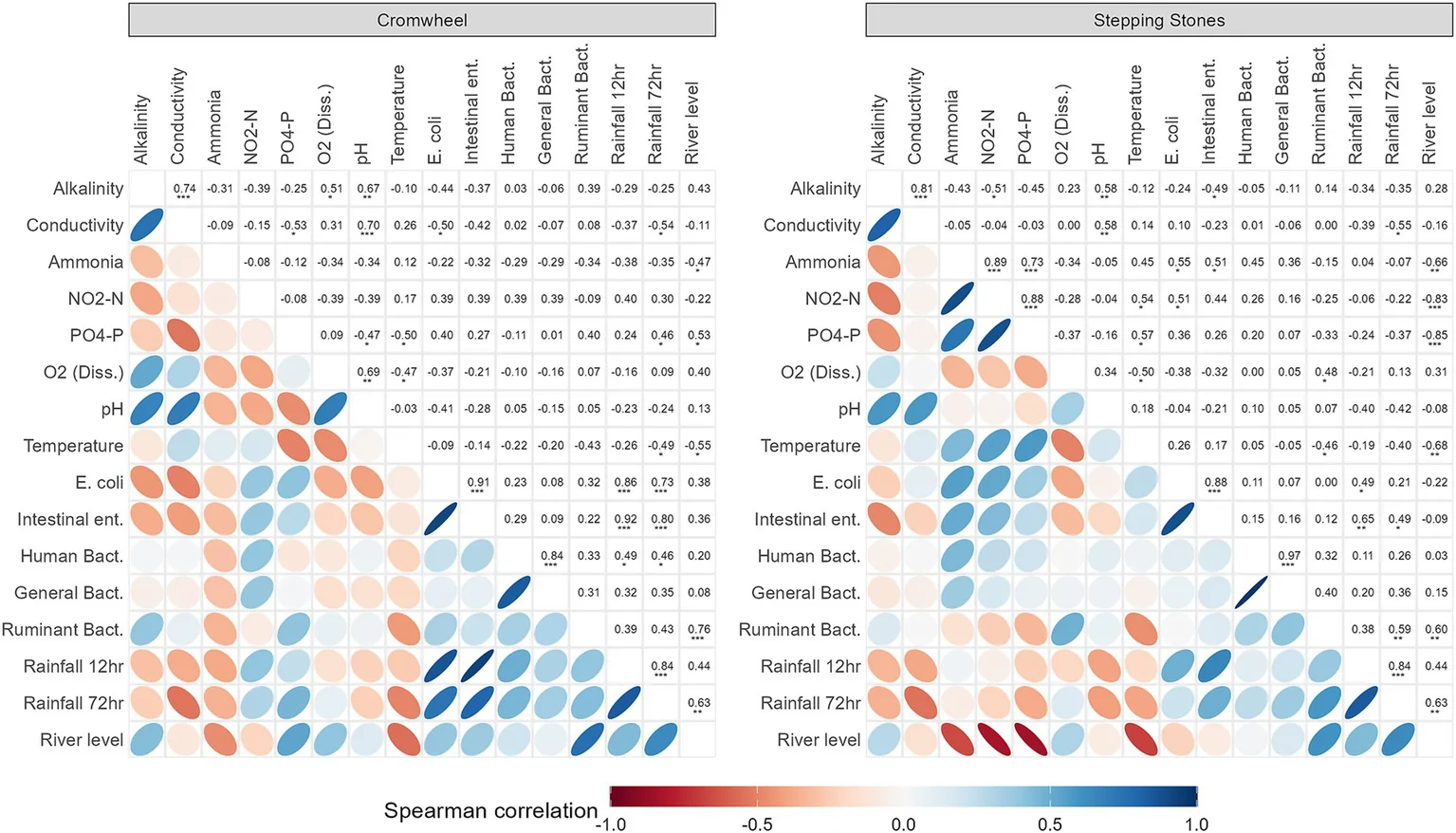
Non-parametric Spearman correlations between physico-chemical, environmental and microbiological parameters using data from both Cromwheel and Stepping Stones independently. Correlation coefficients are shown in the top triangle, and significant correlations are coded with *, with * = *p* ≤ 0.05, ** = *p* ≤ 0.01, and *** = *p* ≤ 0.001.

Non-parametric correlations were obtained independently for both sites (Figure 8). The results for all antecedent rainfall (3, 6, 12, 24 and 72 h are shown in Supplementary Figure S3). Results showed distinct patterns across the two sites. At both Cromwheel and Stepping Stones, alkalinity, pH, and DO were strongly and positively correlated, while nutrients (e.g., ammonia, nitrite and phosphate) were positively associated with each other but negatively with DO. Ammonia was negatively correlated with FIB and the three *Bacteroidales* markers in Cromwheel samples, while at Stepping Stones this parameter was positively correlated with FIB, and general and human markers only.

Rainfall had a stronger positive correlation with nitrate and phosphate at Cromwheel, while at Stepping Stones the correlation was weaker and tended to be negative with the increase of accumulative rainfall. Both FIB analyzed by statutory methods, *E. coli* and IE, have a stronger correlation to rainfall at Cromwheel, particularly at 12 h accumulative rainfall. This correlation with the rainfall tended to be weaker at Stepping Stones, this site had a similar correlation with accumulative rainfall at 12 and 24 h for *E. coli*.

Regarding the river levels, the correlation was positive with FIB and the three *Bacteroidales* markers at Cromwheel. At Stepping Stones, the correlation between the river level and FIB was negative and positive for the three *Bacteroidales* marker, being significantly positively associated with the ruminant marker.

The human and general markers were more strongly correlated with each other than with the ruminant marker. The relationship between rainfall and human marker was slightly stronger at Cromwheel than at Stepping Stones, and higher at 12 and 24 h of accumulative rainfall. The ruminant marker correlated slightly more positively at the Stepping Stones site (except for 12 and 24 h), and at this site correlated strongly with the accumulative rainfall at 72 h.

## 4 Discussion

### 4.1 Water quality monitoring, rainfall and faecal pollution indicators

Based on statutory FIB analysis, both sites fail to meet the minimum standards for safe bathing during the monitored season. While this is expected at the Stepping Stones, located downstream of the STW, the reason for the failure at the Cromwheel is less clear, with potential upstream sources including both livestock and human inputs. Temporal changes in FIB and the human marker, particularly during rainfall events, indicated a strong influence of wastewater infrastructure on microbial loads. This is evident at Stepping Stones, especially on 20th of June (*E. coli* > 100,000 cfu/100 mL), reflecting combined discharges from treated effluent and storm overflows at Ilkley WWTP. Similar patterns were observed at Cromwheel during heavy rainfall (20th June 12th July, and 20th September). At two of these events (20th June and 20th September), rainfall triggered sewage spills from upstream overflows (Rivadale CSO and Addingham PSO), as confirmed by EDM data (Environment Agency, 2025) (Supplementary Figure S1). On the 20th of September, both human and ruminant markers presented similar levels, suggesting mixed contributions from wastewater and agricultural runoff. The 12th of July event was also dominated by human markers, suggesting a sewage source since the sample was taken when the Rivadale CSO was spilling. Outside these events, *E. coli* concentrations remained below 1,000 cfu/100 mL in most samples (16 samples of a total of 19). Overall, MST profiles during extended dry periods (May and early June) were dominated by human markers, most likely reflecting continuous effluent contributions from small upstream WWTPs. However, several samples taken later in the summer after light rainfall events were dominated by ruminant markers.

The FIB levels at Cromwheel had a stronger correlation to rainfall, particularly at 12 h accumulated rainfall, while at Stepping Stones this correlation was stronger at 24 h for *E. coli*. However, the EA, during a monitoring campaign in the River Wharfe (2021–2024) found the strongest relationship to be after 48 h (Environment Agency, 2025). Previous studies in other rivers have shown that precipitation several days prior to sample collection has an impact on microbial concentrations in the samples (Hynes et al., 2024; Peed et al., 2011). Tornevi et al. (2014) showed that heavy rainfall events (exceeding 15 mm in 24 h) were associated with significant increases in microbial concentrations, particularly 48 h after the rainfall event, concluding that rainfall is a major driver of microbial water quality variations. Similarly, Fang et al. (2018) in an urban catchment, highlighted that heavy rainfall led to rapid declines in water quality, often failing to meet recreational quality guidelines, emphasising the need for caution regarding recreational activities during or immediately after rainfall events. For recreational water bodies, normally the advice is not to swim during or within 48 h of a heavy rainfall event in coastal areas (He and He, 2008). However, our study shows that accumulated rainfall at 12 h presented the strongest positive correlation at the Cromwheel site with FIB and human associated faecal markers.

Similarly, the MST results for the three markers increased during rainfall events, yet the contribution of the human-associated markers was higher than that of the ruminant for all samples combined. The Cromwheel was less impacted by faecal pollution in dry weather conditions, but increased during heavy rainfall events, most likely originating from a combination of surface water runoff, spills from CSOs and the Addingham PSO (Supplementary Table S3) and in runoff from agricultural land. A previous study by the EA in the river Wharfe (Environment Agency, 2025) provides evidence for significant mobilisation of FIBs from agricultural land in wet weather from sources higher up in the catchment. Ruminant marker levels were often higher at Cromwheel than at Stepping Stones, particularly in August and on 15th of September. This indicates that agricultural runoff was a key pollution source during moderate rainfall, enough to wash faecal bacteria from land, but not sufficiently intense to cause CSOs and PSOs to spill. This precipitation effect on genetic markers has been reported previously by Vadde et al. (2024), in urban watersheds, where higher levels of the ruminant marker (Rum2Bac) under wet weather, indicated that stormwater runoff contributed to non-point sources of faecal pollution from livestock.

Coincident with rainfall events, higher levels of FIB and potential pathogenic genera such as *Aeromonas* spp., *Shigella* spp., and *Enterococcus* spp. were observed at both sites, but these were clearly higher at Stepping Stones, reinforcing the negative impact of wastewater infrastructure inputs at this site. Sequences associated with *Shigella* and *Enterococcus* in rivers are clearly indicating faecal contamination and a potential risk to human health (Smith et al., 2017). Similarly, the presence of *Aeromonas* spp., while a natural inhabitant of rivers, can become a potential health risk at higher abundance. Several species such as *A. hydrophila* and *A. veronii*, are associated with gastroenteritis, and their presence can affect particularly those with compromised immunity or wounds (Guerra et al., 2022).

The Cromwheel site had intermittently high FIB concentrations, but the overall physico-chemical quality of the water is good when compared with polluted sites in the same river (Karunakaran et al., 2024). The Stepping Stones on the other hand had both high FIB concentrations and poor water quality, with higher orthophosphate and ammonia concentrations caused by the continuous discharge of treated effluent and the intermittent discharge of untreated and diluted storm overflow effluent. Although nutrients and faecal pollution monitoring in rivers are often conducted separately, they are interconnected and pose challenges when they are not investigated together, limiting appropriate solutions for adequate river management. Previous studies have shown that nitrogen and faecal bacteria entered surface waters as co-pollutants (Zimmer-Faust et al., 2025). The levels of ammonia and nitrate were positively correlated with FIB mainly in the Stepping Stones, where ammonia showed a significant positive correlation with the human marker (Figure 8). It is also worth noting that riverbed sediments can provide areas for attachment to microorganisms, acting as long-term reservoirs, where microbes can survive and thrive, and where concentrations of microbes including pathogens can exceed those of the water column (Bai and Lung, 2005). Heavy rainfall can increase river flow and cause sediment to resuspend, thus releasing into the water the stored bacteria once the critical shear stress of the bed is exceeded (Jamieson et al., 2005; Wyness et al., 2019). Concerns regarding FIB-contaminated sediments in the Cromwheel backwater were investigated by the Environment Agency in 2023 (Environment Agency, 2025). The study found that following moderate rainfall events, when a turbid plume was observed, sediment samples exhibited elevated FIB concentrations compared with those in the main river. Sampling sediments was not within the remit of this study, but the risk of resuspension of FIB contaminated riverbed sediments should be considered in future studies to provide a more comprehensive understanding of microbial risks in bathing sites.

### 4.2 ddPCR measurements of faecal markers and source apportionment

Based on our previous study using similar primer-probe combinations and samples from the same river (Karunakaran et al., 2024), the results from this study indicate that ddPCR had better sensitivity in quantifying faecal bacterial markers than the traditional qPCR. The advantages over traditional qPCR (i.e., the ability to perform absolute quantification and directly compare samples across different runs) have also been demonstrated in other studies (Salipante and Jerome, 2019; Staley et al., 2018; Tiwari et al., 2022; Wang et al., 2022). Molecular methods and in particular ddPCR offer a faster and more efficient way to quantify pollution, thus aiding management of bathing sites in response to pollution events.

Whereas Stepping Stones samples were dominated by human markers, the relative contribution of human and ruminant markers at the Cromwheel varied over time. During high rainfall events (20th June and 12th July), Cromwheel samples were dominated by human markers. The 20 June event coincided with a prolonged untreated effluent spill from Addingham PSO upstream lasting 400 min, (Supplementary Table S3). While the 12th July event, although not linked to Addingham, may be associated with a spill from Rivadale CSO. The 20th September event did correspond to a long duration spill (over 265 min) from Addingham PSO. However, ruminant levels for this sample were similar to those for human markers suggesting that the high FIB concentrations at this time likely result from a combination of sewage discharges and agricultural runoff. This pattern may suggest earlier rainfall in the upper catchment, mobilising livestock-derived contamination before triggering CSO spills downstream. At the Cromwheel, the proportions from human and ruminant sources were similar (Figure 4), yet samples taken in August and 15th September were high in ruminant numbers.

Different studies have been successful in investigating sources of faecal pollution in freshwater ecosystems using different genetic markers (Ballesté et al., 2020; Tiwari et al., 2022; Vadde K. K. et al., 2019). In this study, the level of the general marker was higher, and the overall contribution to faecal pollution was human-associated at both sites, except for the Cromwheel in August where the ruminant indicator presented higher levels than the human (Figure 4). On average, samples at both sites presented low levels of ruminant-associated markers, however the contamination due to other animals was not considered, yet by amplicon sequencing (Figure 6) we found a very small percentage of bacteria previously related to avian gut microbiota, *Bacteroides caecigallinarum* (Saputra et al., 2015). When data from both sites were considered together, cumulative antecedent rainfall had a significant positive correlation with the concentrations of *E. coli* and IE, and MST markers, particularly with the human marker, and to a lower extent the general marker. This again indicates that the main source of pollution affecting the sampling sites was wastewater from urban water infrastructure. This was expected at the Stepping Stones site, but it was less expected at the Cromwheel (main designated bathing water site), where two of the three storm event samples recorded in 2023 were dominated by human markers.

This site has failed to meet a safe standard for bathing each year since its designation in 2020 (Environment Agency, 2020), likely due to high concentrations of faecal bacteria washed during rainy events from both agricultural and urban wastewater sources. Our results suggest that agricultural sources were less important in 2023 and that current efforts by the local water company to control storm overflows from upstream CSOs and PSOs may thereby lead to significant improvements in public health safety at the bathing site. However, stormwater control measures alone cannot address all infrastructure-related sources of river contamination (Carson Liam et al., 2024; Whelan et al., 2022). In rural areas, additional inputs such as private wastewater systems (e.g., septic tanks) and agricultural sources remain significant contributors, particularly during wet weather (Battarbee et al., 2021; Environment Agency, 2025). EA monitoring evidence (Environment Agency, 2025) suggests that the balance between human and ruminant sources of contamination may vary significantly from event to event depending on the location, intensity, amount and length of rainfall episodes in the catchment. Therefore, improvement in wastewater treatment in urban areas upstream of designated bathing water sites as well as control of livestock in agricultural catchments are both needed to protect recreational users. Such improved management is particularly urgent considering the increasing pressures from climate change, which are expected to exacerbate rainfall variability and CSOs spills (Johnson et al., 2009; Whitehead et al., 2009).

### 4.3 Microbial community structure: indicators of faecal pollution, potential and opportunistic pathogens (OPs)

Amplicon sequencing was able to identify pollution trends and potential microbial risks to a level of detail that the traditional FIB cannot. Clear differences in the microbial community structure were found between the two sites, downstream and upstream of the WWTP (Figure 5). The downstream site, Steppings Stones, was characterized by higher levels of *Clostridia* and *Bacilli*, although the levels of these increased at both sites in response to heavy rainfall events. One of the *Clostridia* species, *Clostridium perfringens* is an indicator of faecal pollution, and unlike other bacterial indicators such as *E. coli*, can form spores that can persist longer in the environment, making it useful for assessing past or intermittent faecal contamination (Suzuki et al., 2021). *C. perfringens* was found more frequently in the Stepping Stone samples (Supplementary Table S2). The distinctive community fingerprint coincident with episodes of faecal pollution (coincident with FIB spikes) included *Bacteroides*, *Faecalibacterium*, *Bacilli*, *Eubacterium*, *Aeromonas* and *Clostridium* (Gonçalves Pessoa et al., 2019; Janda and Abbott, 2010) (Supplementary Table S2). Therefore, changes in bacterial community structures can be a good indicator of perturbation, and have been successfully used in other studies to identify faecal contamination in freshwater (Unno et al., 2018; Vadde K. et al., 2019). DNA sequencing helped to detect pollution signatures at species level (Figure 6) from faecal sources such as *Faecalibacterium prausnitzii* and *Eubacterium rectale*, both human specific bacteria, that could be used as an alternative faecal indicator marker (Fitzgerald et al., 2018; Schwiertz et al., 2000).

Microbial community profiling also provided information on the presence of pathogenic bacteria and OPs, which should be monitored in relation to public health protection (Pereira et al., 2018). In this study, we found sequences assigned to *Shigella* spp., *Enterococcus faecalis*, *Legionella pneumophila*, and several species of *Mycobacteria* and S*treptococcus* that are pathogenic or opportunistic pathogens (Arias and Murray, 2012; Fields et al., 2002; Mena and Gerba, 2009). These were more abundant in the Stepping Stones site, suggesting once again that improvements in wastewater infrastructure are needed to protect sites downstream of WWTPs, where faecal bacteria are introduced via treated effluent. At the bathing site, the percentage of sequences associated to pathogenic bacteria were very low [<5% per species (Supplementary Table S2)], and only certain bacteria such as *Acinetobacter* spp. *Aeromonas* spp. *Leptospira* spp. (Pompilio et al., 2019), *Legionella pneumophila*, *Mycobacterium llatzerense* and *Mycobacterium neglectum* showed relative abundance values > 5%. The presence of these bacteria at the bathing site requires further investigation to fully assess their viability and the actual risk of infection. Nevertheless, their detection indicates that enhancements in site management may be needed to ensure safe recreational use.

Understanding the potential health risks associated with faecal pollution in bathing sites is crucial, yet it should be noted that the presence of pathogenic taxa based only on DNA sequence amplicon may not always correlate with actual public health risks, as for example the presence of virulence genes should also be considered. To increase pollution protection during dry weather, improving the performance of WWTP by implementing disinfection processes would be a suitable measure. To prevent human infections through contaminated water, during wet weather events, public awareness of potential hazards should be raised together with measures to avoid CSOs spills. The approach presented here is useful to inform risk assessment models, supporting water safety plans in recreational sites.

## 5 Conclusion

The use of traditional FIB, MST and amplicon sequencing indicated that sources of pollution were mainly human with peaks associated with wet weather events at both sites. Human sources are to be expected at the Stepping Stones site, immediately downstream of the STW, but human MST markers were overall higher than ruminants at the Cromwheel, the main bathing water site, especially so after heavy rainfall events. However, agricultural sources of pathogenic bacteria are nevertheless important in wet weather conditions and these sources as well as wastewater infrastructure sources may also need to be controlled if the Cromwheel site is to achieve a “sufficient” classification for bathing under the UK Bathing Water Regulations.

The findings of this study highlight the need for more frequent sampling and the application of advanced molecular methods, beyond traditional culture-based techniques, to accurately identify sources of pollution relevant to recreational water quality management. This aligns with the recast EU Urban Wastewater Directive (EU) 2024/3019, which recognizes the need for more precise water quality monitoring, particularly for recreational and bathing waters. Advancements in molecular technologies and a frequent and robust water quality monitoring approach are key to informing decision-making in water resource management in recreational bathing sites.

## Data Availability

The sequencing data presented in this study is publicly available. The data can be found in NCBI Sequence Read Archive (SRA) via accession number PRJNA1414228.

## Footnotes

1
www.ncbi.nlm.nih.gov

## Supplementary material

SUPPLEMENTARY FIGURE S1 Yorkshire Water (YW) spills events data related to Combined Sewer Overflow (CSO) and Sewage Pumping Station (SPS) over the sampling season.

SUPPLEMENTARY FIGURE S2 Heatmaps: Hierarchical clustering using relative abundance for both sites at genera level was performed using Bray–Curtis dissimilarity and average linkage (UPGMA).

SUPPLEMENTARY FIGURE S3 Non-parametric Spearman correlation between physico-chemical, environmental and microbiological parameters using data from both Cromwheel and Stepping Stones independently. Showing correlations with antecedent rainfall at 3, 6, 12, 24, 48 and 72 h.

SUPPLEMENTARY TABLE S1 Sequences of primers and probes used for the Microbial Source Tracking analysis using ddPCR.

SUPPLEMENTARY TABLE S2 List of relative abundance of pathogenic bacteria and opportunistic pathogenic bacteria found at both sites, including references to available literature.

SUPPLEMENTARY TABLE S3 Spills from Combined Sewer Overflows (CSO) and Pumping Station Overflow (PSO) in the monitored area.
