# Supplementary material for: Rainfall-driven dynamics of faecal pollution in river bathing sites: coupling hydrological events, and DNA-assisted tracking to improve pollution management in recreational waters
Source: Front Microbiol. 2026 Aug 10;17:1796625. doi: 10.3389/fmicb.2026.1796625 (PMC13501524; doi:10.3389/fmicb.2026.1796625)
Supplement: Supplementary file 1 [file Image_2.pdf]

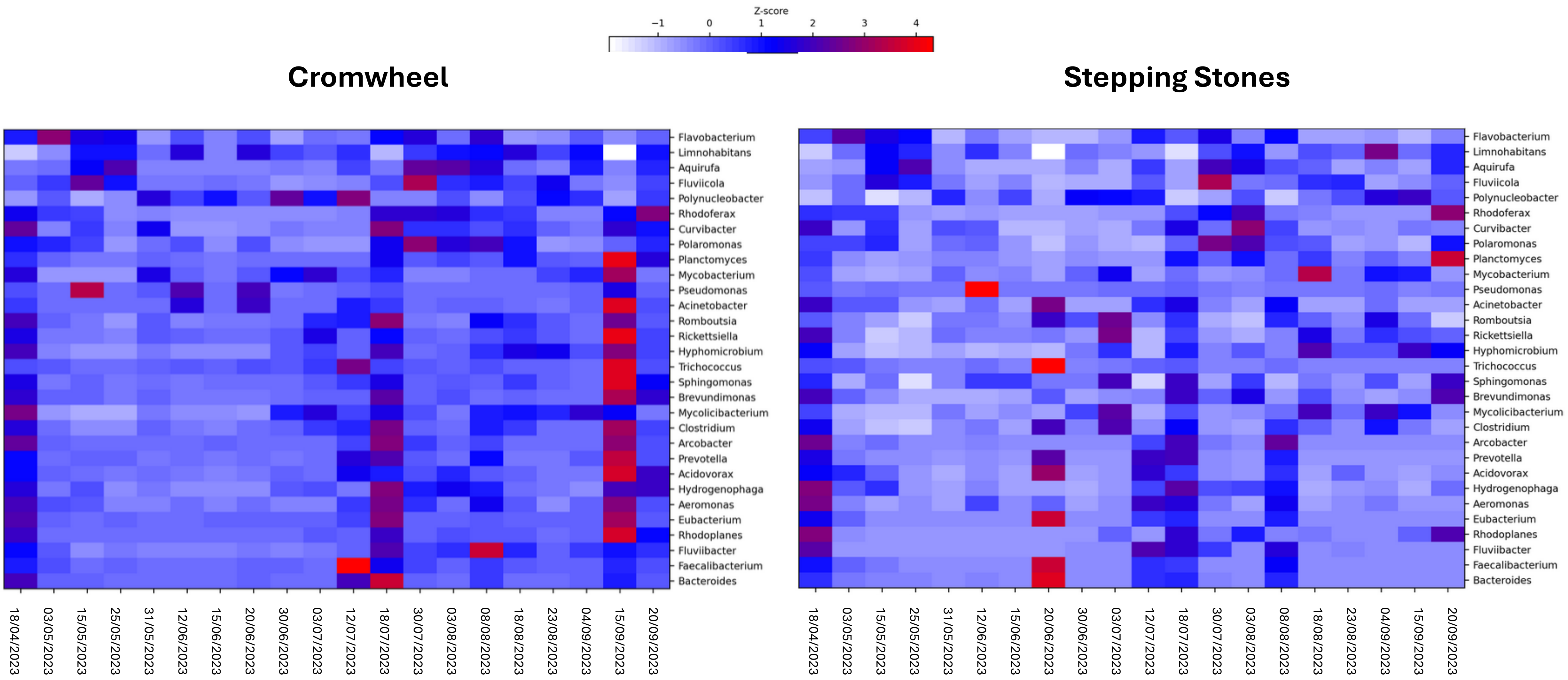
Z-score
−1
0
1
2
3
4
Cromwheel
Stepping Stones
Flavobacterium
Limnohabitans
Aquirufa
Fluviicola
Polynucleobacter
Rhodoferax
Curvibacter
Polaromonas
Planctomyces
Mycobacterium
Pseudomonas
Acinetobacter
Romboutsia
Rickettsiella
Hyphomicrobium
Trichococcus
Sphingomonas
Brevundimonas
Mycolicibacterium
Clostridium
Arcobacter
Prevotella
Acidovorax
Hydrogenophaga
Aeromonas
Eubacterium
Rhodoplanes
Fluviibacter
Faecalibacterium
Bacteroides
18/04/2023
03/05/2023
15/05/2023
25/05/2023
31/05/2023
12/06/2023
15/06/2023
20/06/2023
30/06/2023
03/07/2023
12/07/2023
18/07/2023
30/07/2023
03/08/2023
08/08/2023
18/08/2023
23/08/2023
04/09/2023
15/09/2023
20/09/2023
